# Correction to “implementing pain, agitation, delirium, and sleep deprivation protocol in critically ill patients: A pilot study on pharmacological interventions”

**DOI:** 10.1111/cts.70068

**Published:** 2024-11-04

Luetrakool P, Taesotikul S, Susantitapong K, Suthisisang C, Morakul S, Sutherasan Y, Tangsujaritvijit V, Dilokpattanamongkol P. Implementing pain, agitation, delirium, and sleep deprivation protocol in critically ill patients: A pilot study on pharmacological interventions. *Clin Transl Sci*. 2024 Mar;17(3):e13739. doi: 10.1111/cts.13739. PMID: 38421247; PMCID: PMC10903435.

On page 8, under the section titled “FUNDING INFORMATION”, the sentence “The authors confirm that this research received no external funding. The study was conducted without financial support, and the authors have no financial interests or affiliations that could be perceived as a conflict of interest in connection with this work.” were incorrect. This should have read: “This research project is supported by Mahidol University. The authors have no financial interests or affiliations that could be perceived as a conflict of interest in connection with this work.”

We apologize for this error.

